# Effect of Removing Superior Spikelets on Grain Filling of Inferior Spikelets in Rice

**DOI:** 10.3389/fpls.2016.01161

**Published:** 2016-08-05

**Authors:** Cuicui You, Honglei Zhu, Beibei Xu, Wenxiao Huang, Shaohua Wang, Yanfeng Ding, Zhenghui Liu, Ganghua Li, Lin Chen, Chengqiang Ding, She Tang

**Affiliations:** ^1^College of Agronomy, Nanjing Agricultural UniversityNanjing, China; ^2^Key Laboratory of Crop Physiology Ecology and Production Management, Ministry of AgricultureNanjing, China; ^3^Jiangsu Collaborative Innovation Center for Modern Crop ProductionNanjing, China

**Keywords:** rice, superior spikelet removal, plant hormones, inferior spikelets, grain filling, sucrose to starch metabolism, enzyme activity

## Abstract

Large-panicle rice cultivars often fail to reach their yield potential due to the poor grain filling of inferior spikelets (IS). Thus, it is important to determine the causes of poor IS grain filling. In this study, we attempted to identify whether inferior grain filling of large panicles is restricted by superior spikelets (SS) and their physiological mechanism. SS were removed from two homozygous japonica rice strains (W1844 and WJ165) during flowering in an attempt to force photosynthate transport to the IS. We measured the effects of SS removal on seed setting rate, grain weight, grain filling rate, sucrose content, as well as hormone levels, activities of key enzymes, and expression of genes involved in sucrose to starch metabolism in rice IS during grain filling. The results showed that SS removal improved IS grain filling by increasing the seed setting rate, grain weight, sucrose content, and hormone levels. SS removal also enhanced the activities of key enzymes and the expression levels of genes involved in sucrose to starch metabolism. These results suggest that sucrose and several hormones act as signal substances and play a vital role in grain filling by regulating enzyme activities and gene expression. Therefore, IS grain filling is restricted by SS, which limit assimilate supply and plant hormones, leading to poor grain filling of IS.

## Introduction

Rice is one of the most important food crops in the world, and its consumption is expected to increase with further population growth; therefore, improving the output per unit area is the only option for producing more rice on a limited land area (Khush, 2005). In order to increase rice yield, many scientists involved in genetic breeding are attempting to expand sink capacity by increasing the number of grains per panicle, cultivating large-panicle strains of rice (Kato et al., 2007). For example, the capacities of a new type of rice from the International Rice Research Institute and a heavy panicle hybrid rice used for rice production have been significantly improved (Peng et al., 1999). These cultivars, however, do not always produce higher yields due to low seed setting rate and grain weight of inferior spikelets (IS) (Zhang et al., 2009; Yang and Zhang, 2010). Therefore, improvements in IS grain filling would have a significant impact on the yield potential of large-panicle rice.

There are many possible explanations for poor grain filling and low grain weight of the IS, including low enzyme activity in the conversion of sucrose to starch (Tang et al., 2009; Yang and Zhang, 2010; Wang et al., 2015), hormone imbalances (Tang et al., 2009; Zhang et al., 2009; Wang et al., 2015), assimilate transportation barriers (Yang and Zhang, 2010; Fu et al., 2011), and the differential expression of genes associated with cell growth and signal transduction (Mohapatra and Panigrahi, 2011). However, whether the lack of assimilate supply is a limiting factor for IS grain filling is still unknown. Many researchers have found that at the early stage of the grain filling period, the concentrations of soluble carbohydrates in the IS are higher than those in the superior spikelets (SS), suggesting that assimilate supply is not the main reason for poor IS grain filling (Mohapatra et al., 1993; Yang and Zhang, 2010). However, Ishimaru et al. (2005) and Tang et al. (2009) found that the IS sucrose concentration was lower than that in the SS. Moreover, other research has suggested that slow grain filling and low grain weight of IS were mainly due to limitations in the carbohydrate supply. After the heading stage, 60–80% of the nutrients needed for grain filling come from leaf photosynthesis. The photoassimilate is always preferentially supplied to the SS; therefore, when photosynthetic products are in short supply, the grain filling rate of the IS may be reduced or incomplete (Wang, 1981; Murty and Murty, 1982). Zhu et al. (1988) discovered that increasing the assimilate supply could restore once-stagnant IS grain filling. Previous studies have often used leaf- and flower-thinning methods to regulate the source–sink balance (Seo, 1980; Xu et al., 2005). For example, Xu F. et al. (2013) removed the middle and upper spikelets of rice and found that the grain weight and the seed setting rate of the remaining IS increased significantly. However, Kato (2004) found that removing part of the spikelets did not significantly improve IS seed setting. For this reason, the authors believed that the poor grain filling in IS was not due to limited resources. Besides, most previous studies focus only on the relationship between spikelets thinning treatment and grain weight, but little attention to its related mechanism. Therefore, the question of whether increasing assimilate supply will improve the grain filling of IS and the underlying physiological mechanism for this remains unanswered.

Many reports have indicated that carbohydrates and plant hormones play vital roles in the regulation of grain filling (Liang et al., 2001; Yang et al., 2001, 2006). It is reasonable to assume therefore that the development of IS may be regulated by the assimilate supply and plant hormones after SS are removed. It is believed that plant hormones, including zeatin riboside (ZR), indole-3-acetic acid (IAA), and abscisic acid (ABA), are closely associated with grain development (Bangerth, 1989; Wobus and Weber, 1999), regulating the sink size of grains by mediating the division and enlargement of endosperm cells during the early phase of seed filling (Brenner and Cheikh, 1995; Kende and Zeevaart, 1997; Hansen and Grossmann, 2000). IAA and ABA are also thought to be involved in the mobilization and accumulation of assimilates in seeds (Seth and Waering, 1967; Singh and Gerung, 1982; Kato et al., 1993). The relationship between plant hormones and grain filling in rice has been intensively investigated (Kato and Takeda, 1993; Yang et al., 2001). Yang et al. (2001) reported that during grain filling, especially at early stages, the ZR, IAA, and ABA contents in SS are consistently higher than those in IS. Wobus and Weber (1999) showed that plant hormones play an important role in determining the variation in grain development among spikelets in a panicle. However, it is unclear if the levels and dynamics of hormone content in the developing grains are related to the source–sink relationship.

Grain filling is actually a process of starch biosynthesis and accumulation (Yoshida, 1972). Grain filling materials come from carbohydrates stored in the stem and sheath before heading and from photosynthetic products after heading (Liang et al., 1994; Xie et al., 2001). These assimilates are transported from the source to the grain mainly in the form of sucrose and are converted to starch through a series of enzymatically catalyzed reactions. Among these enzymes, sucrose synthase (SuSase), ADP-glucose pyrophosphorylase (AGPase), soluble starch synthase (SSS), and starch branching enzyme (SBE) are considered to play key roles in the process of grain filling (Mizuno et al., 1992; Stark et al., 1992; Keeling et al., 1993; Liang et al., 2001). Numerous studies have shown that there are many genes involved in controlling the synthesis starch from sucrose, including *SuS2*, *SuS3*, *SuS4*, *AGPS1*, *AGPL1*, *AGPL2*, *SSSI*, *SSSII-3*, *SSSIII-2*, *SBEI*, *SBEIII*, and *SBEIV* (Miyazawa et al., 1999; Rook et al., 2001; Ohdan et al., 2005). Recent studies have clarified the relationships between starch synthesis, the activities of key enzymes, and the expression levels of genes involved in the sucrose to starch conversion (Zhu et al., 2011; Wang et al., 2014). Yet, the effect of SS removal on the expression levels and activities of these key starch synthesis enzymes and how these interact with IS grain filling remains unclear.

The objectives of this study were to investigate whether IS grain filling of large panicles is limited by SS through removal of some SS and examination of subsequent changes in seed setting rate, grain weight, grain filling rate, sucrose content, hormone levels, activities of key enzymes, and expression levels of genes involved in sucrose to starch metabolism in the IS during the grain filling period. We also sought to determine whether these changes were correlated with IS post-anthesis development in two homozygous large-panicle japonica strains.

## Materials and Methods

### Plant Materials

This experiment was conducted in 2014 at the Danyang Experimental Base of the Nanjing Agricultural University, Jiangsu Province, China (31°54′31″N, 119°28′21″E) during the rice growing season. In order to analyze inferior grain filling at the molecular level, the experiment was conducted using two homozygous large-panicle japonica rice strains, W1844 and WJ165, from the State Key Laboratory of Rice Genetics and Germplasm Innovation, Nanjing Agricultural University. The agronomic traits of the two rice strains are shown in **Table 1**. Grains per panicle of the two rice strains are above 250, which is typical of heavy panicle materials. Compared with W1844, WJ165 exhibits higher values for plant height, panicle length, and grain weight, but lower grain growth density and seed setting rate. Seedlings were field-grown and transplanted 22 days after sowing (May 26) at a hill spacing of 13.3 cm × 30 cm with three seedlings per hill. Plot dimensions were 5 m × 10 m. Each rice strain was grown in three replicate plots in a completely randomized block design. The soil at the experimental site was clay loam. Nitrogen application throughout the whole growing season was 280 kg ha^-1^. The amount of nitrogen fertilizer applied was converted into urea according to the nitrogen content, and the application ratio of base fertilizer:panicle fertilizer was 5:5. The base fertilizer was applied before transplanting, and the panicle fertilizer was applied when the leaf-age remainder was 3.5. The heading date (50% plants) for W1844 and WJ165 was on September 4–6, and plants were harvested on November 7–9. Cultivation and management measures were applied according to the technical requirements of the local field. The average air temperatures during the rice grain filling period as measured at a weather station close to the experimental site are shown in **Supplementary Figure S1**.

**Table 1:** Agronomic traits of the japonica rice materials.

Materials	Days from sowing to maturity	Plant height (cm)	Panicle length (cm)	Grain growth density	Grains per panicle	1000-grain weight (g)	Setting rate (%)
W1844	148	102.5 ± 4.79 b	15.9 ± 2.26 b	16.7 ± 0.98 a	265.0 ± 5.62 a	24.2 ± 0.32 b	89.3 ± 4.32 a
WJ165	153	134.7 ± 7.77 a	21.8 ± 1.51 a	11.8 ± 0.13 b	257.3 ± 2.79 a	31.1 ± 0.23 a	86.7 ± 2.06 b


*Grain growth density = grains per panicle/panicle length. The different lowercase letters labeled after the data from the same character under the different variety indicate significant differences at the 0.05 level.*

### Experimental Design

At about 10 am on September 1, 2014, many panicles started heading and the spikelets at the top of the panicle began opening in both rice strains used in this experiment. A total of 600 plants with a similar growth patterns that flowered on the same day were labeled. On September 5–6, most labeled panicles had withdrawn from the flag leaf sheath completely, and spikelet-thinning treatments were performed according to the following protocol. In total, there were three treatment groups: group 1 was the control group with no spikelet thinning (labeled T0), group 2 plants had the upper 1/3 of spikelets removed (T1), and group 3 had the upper 2/3 of spikelets removed (T2). Spikelet thinning involved removal of the primary branch. The primary branches from the whole panicle were equally divided into three parts: upper, middle, and lower parts. If the number of primary branches could not be divided equally, a number of spikelets equal to the integer of the average branch number was included in each of the upper and lower parts, and the remaining branches were included in the middle part. SS were considered to be the grains on the three primary branches on the upper part of the panicle, while medium spikelets (MS) were defined as the grains on the three primary branches in the middle part of the panicle, and IS were the grains on the three second branches in the lower part.

### Sampling and Measurement

#### Determination of the Grain Filling Rate

We sampled 50 tagged panicles from each plot every 5 days from anthesis to maturity, and froze 3/5 of sampled grains in liquid nitrogen for 1 min before storing at -80°C. These were used for determination of plant hormone levels, as well as the activities and gene expression levels of the key enzymes SuSase, AGPase, SSS, and SBE. The remaining grains were deactivated at 105°C for 0.5 h and dried at 80°C to a constant weight. They were then weighed and dehulled to determine the grain dry weights (DWs) and sucrose contents. The processes of grain filling were fitted with Richards’s growth equation (Richards, 1959):

$$W=\frac{A}{{\left(1+Be^{-kt}\right)}^{1/N}}$$

The grain filling rate (*R*) was calculated as the derivative of the Eq. 1.

$$R=\frac{Ak{Be}^{-kt}}{{N\left(1+{Be}^{-kt}\right)}^{(N+1)/N}}$$

where *W* is the grain weight (mg), *A* is the final grain weight (mg), *t* is the time after anthesis (days), and *B*, *k*, and *N* are coefficients established from the regression of the equation.

#### Determination of Sucrose Content

The sucrose determination method was modified from the method of Yoshida et al. (1976). Samples were dried in an oven, ground to a fine powder, and sieved through a 100 mesh sieve. The ground sample (0.1 g) was placed into a 15 mL centrifuge tube with 8 mL of 80% ethanol, and the samples were placed into an 80°C water bath for 30 min. After cooling, the sample was centrifuged at 5000 rpm for 15 min. The extraction process was repeated three times, and the supernatants were combined and distilled water was added up to 50 mL. Took a new 15 mL centrifuge tube, 0.9 mL of the extract and 0.1 mL of 2 M NaOH was added and then the centrifuge tube was placed into a boiling water bath for 30 min. After cooling, 1 mL of 0.1% resorcinol (0.1 g resorcinol dissolved in 100 mL distilled water) and 3 mL of 10 M HCl were added. The sample was placed into an 80°C water bath for 30 min, and colorimetric determination was performed at 500 nm.

#### Extraction and Determination of Hormone Levels

The levels of ZR, IAA, and ABA were determined by Zoonbio Biotechnology Co., Ltd, and the methods were modified from those described by Pan et al. (2002). Approximately 0.5 g dehulled grains were ground in a pre-cooled mortar that contained 5 mL extraction buffer composed of isopropanol/hydrochloric acid. The extract was shaken at 4°C for 30 min. Then, 10 mL dichloromethane was added, and the sample was shaken at 4°C for 30 min and centrifuged at 13,000 rpm for 5 min at the same temperature. We then extracted the lower, organic phase. The organic phase was dried under N_2_ and dissolved in 150 μL methanol (0.1% methane acid) and filtered with a 0.22-μm filter membrane. The purified product was then subjected to high-performance liquid chromatography-tandem mass spectrometry (HPLC-MS/MS) analysis. HPLC analysis was performed using a ZORBAX SB-C18 (Agilent Technologies) column (2.1 mm × 150 mm; 3.5 mm). The mobile phase A solvents consisted of methanol/0.1% methanoic acid, and the mobile phase B solvents consisted of ultrapure water/0.1% methanoic acid. The injection volume was 2 μL. MS conditions were as follows: the spray voltage was 4500 V; the pressure of the air curtain, nebulizer, and aux gas were 15, 65, and 70 psi, respectively; and the atomizing temperature was 400°C.

#### Determination of the Activities of Key Enzymes Involved in Sucrose to Starch Conversion

Activities of SuSase, AGPase, SSS, and SBE in grains were determined according to the method described by Nakamura et al. (1989). A total of 30 sampled grains were dehulled and homogenized with a pestle in a pre-cooled mortar containing 5 mL of 50 mM 4-(2-hydroxyethyl)-1-piperazineethanesulfonic acid (HEPES)–NaOH frozen extraction buffer [pH 7.5, including 10 mM MgCl_2_, 2 mM ethylenediaminetetraacetic acid (EDTA), 50 mM 2-mercaptoethanol, 12.5% glycerol, and 5% polyvinylpyrrolidone-40 (PVP-40)] and kept at 0°C. After being filtered through four layers of cheesecloth, the homogenate was centrifuged at 15,000 *g* for 15 min at 4°C, and the supernatant of the crude enzyme extract was used directly for the enzyme assay.

#### RNA Extraction and Semi-quantification Analysis

According to previous research (Takashi et al., 2005; Tatsuro et al., 2008; Wang et al., 2015), we believe that the following genes are involved in the sucrose to starch conversion process and are closely related to grain filling in rice: *SuS2*, *SuS3*, *SuS4*, *AGPS1*, *AGPL1*, *AGPL2*, *SSSI*, *SSSII-3*, *SSSIII-2*, *SBEI*, *SBEIII*, and *SBEIV*. We determined the gene transcription levels of these genes through RNA extraction, cDNA synthesis, and quantitative real-time polymerase chain reaction (qRT-PCR). Total RNA was isolated from rice grain samples following the RNA extraction kit manual (Tiangen, DP432). RNA quality was assessed using agarose gel electrophoresis and was deemed sufficient when two clear bands for 18S and 28S rRNAs were visualized (Nolan et al., 2006). RNA reverse transcription was performed using the Prime Script^TM^ RT Reagent Kit (Takara, Code No. RR037A) according to the manufacturer’s instructions. Transcript levels of the genes were measured by qRT-PCR using an ABI 7300 Real Time PCR System with SYBR Green (Takara, Code No. RR420A). The gene accession numbers and gene-specific primer pairs used for qRT-PCR are listed in **Supplementary Table S3**.

#### Determination of Pollen Activity

Pollen activity was determined using the “iodine-potassium iodide (I-KI) solution staining” method of Zhang et al. (2007). Panicles, with spikelets in bloom, were taken back to the laboratory, anthers removed, placed on a glass slide, and mashed in two to three drops 1% I-KI solution (1 g KI dissolved in 5–10 mL of distilled water in which 0.5g I_2_ was dissolved and made up to 100 mL with distilled water). Total pollen number and fertile pollen number were recorded from each of three randomly selected horizons. Fertile pollen grains were stained blue, while aborted pollen grains were stained yellowish-brown. This was repeated three times, and the pollen activity was calculated according to the following formula: Pollen activity = fertile pollen number/total pollen number.

#### Yield Performance

At maturity, approximately 250 tagged panicles from each treatment were harvested. The SS, MS, and IS were collected from the T0 group, the MS and IS were collected from the T1 group, and the IS were collected from the T2 group. The samples were naturally dried and the 1000-grain weight was measured.

At maturity, approximately 30 tagged panicles from each treatment were sampled from among the complete panicles with no grain loss, and the seed setting rate was determined using the method of Kobata et al. (2010). The grain was poured into 70%-ethanol solution, and after 2 h, plump grains were those that had sunk to the bottom, while empty and blighted grains were those floating on the water. This was repeated three times, and the seed setting rate was calculated according to the following formula: Seed setting rate = plump grain number/total grain number.

### Statistical Analysis

Statistical analysis of the date was performed using Microsoft Excel 2003 and SPSS 16.0.

## Results and Analysis

### Grain Weight and Seed Setting Rate

There were significant differences in grain weights and seed setting rates among SS, MS, and IS of strains W1844 and WJ165. The SS exhibited the highest grain weight and seed setting rate, followed by MS, with IS exhibiting the lowest values (**Table 2**). The grain weights and seed setting rates of the MS and IS in the T1 group and of the IS in the T2 group were elevated compared to those of the T0 group. Under T2 treatment, seed setting improved considerably; therefore, we focused on the effect of T2 treatment on IS grain filling. For strain W1844, under T2 treatment, the seed setting rate of IS was 90.15%, about four percentage points lower than that of SS in the T0 group. This difference was significant, indicating that pollen activity in IS was still lower than that of SS under T0. However, fertilized IS were well developed, with grain weights reaching values similar to those of SS in the T0 group. In contrast, strain WJ165 exhibited the opposite pattern. Under T2 treatment, the seed setting rate of IS was higher and the grain weight was lower than those of SS in the T0 group, indicating that the grain filling of fertilized grain was poor. Overall, compared with IS in the T0 group, differences in seed setting rate and grain weight between IS in the T2 group and SS in the T0 group were small, demonstrating that SS removal significantly improves the grain weight and seed setting rate of IS.

**Table 2:** Grain weight and seed setting rate under different treatments.

Materials	Treatment	Grain weight (mg/grain)			Seed setting rate (%)		
			
		Superior	Medium	Inferior	Superior	Medium	Inferior
W1844	T0	27.11 a	24.62 c	22.73 d	94.07 a	89.45 b	84.34 c
	T1	–	26.19 b	26.01 b	–	92.59 a	88.92 b
	T2	–	–	27.05 a	–	–	90.15 b
WJ165	T0	32.93 a	29.47 c	26.67 d	88.67 b	86.33 c	84.14 d
	T1	–	31.23 b	28.80 c	–	88.12 b	86.17 c
	T2	–	–	30.37 bc	–	–	90.69 a


*T0, control group without any treatment; T1, top 1/3 of the spikelets were removed; T2, top 2/3 of the spikelets were removed; –, the spikelets that were removed. The different lowercase letters labeled after the data from the same character under the same variety indicate significant differences at the 0.05 level.*

### Sucrose Content in Developing Grains

**FIGURE 1:**
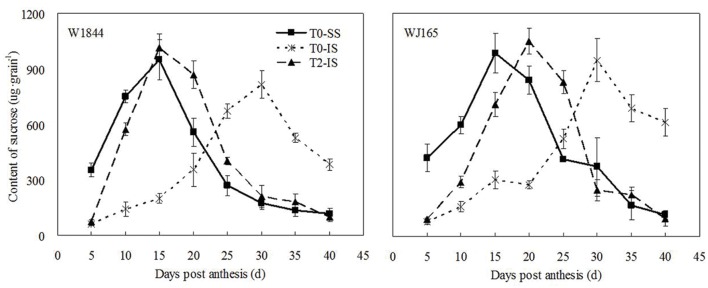
**Sucrose content of SS and IS of rice during grain filling.** T0 and T2 represent control group without any treatment and top 2/3 of the spikelets were removed. Vertical bars represent mean values ± SE (*n* = 3).

**Figure 1** illustrates changes in the sucrose contents of both SS and IS from strains W1844 and WJ165 during grain filling. During the early filling stage, the SS sucrose content was significantly higher than that of IS and reached its the highest value after 15 days post-anthesis (DPA), and then decreased. In contrast, in IS, the sucrose content was very low during the early filling stage and increased slowly after 10 DPA, peaking at 30 DPA. After SS was removed, sucrose content of IS increased significantly, and exceed the highest value of SS in T0 treatment after 15 DPA (W1844) and 20 DPA (WJ165), respectively, and decreased dramatically thereafter. In short, after SS was removed, the variation pattern of IS sucrose content was similar with that of SS in T0 and this suggesting that IS obtained sufficient carbohydrates after SS were removed.

### Grain Filling of Superior and Inferior Spikelets

**FIGURE 2:**
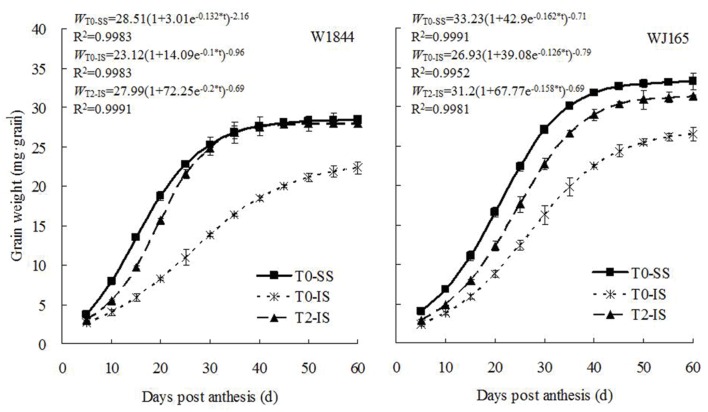
**Grain weight of SS and IS of rice during grain filling.** T0 and T2 represent control group without any treatment and top 2/3 of the spikelets were removed. Vertical bars represent mean values ± SE (*n* = 3).

The dynamics of changes in grain weight and the results from the Richards equation simulation during the grain filling period are shown in **Figure 2**. The grain weight of IS was consistently lower than that of SS during the whole filling period. IS subjected to T2 treatment exhibited higher grain weights, reaching a level similar to that of SS in the T0 group 30 days after flowering in strain W1844. In strain WJ165, IS grain weight increased under T2 treatment but never reached a level similar to that of SS in the T0 group. Overall, however, T2 treatment appeared to significantly improve IS grain weight.

**FIGURE 3:**
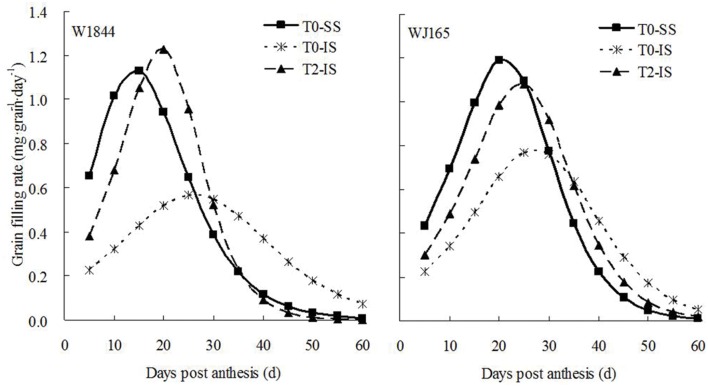
**Grain filling rate of SS and IS of rice during grain filling.** T0 and T2 represent control group without any treatment and top 2/3 of the spikelets were removed.

Initial and maximum IS grain filling rates were consistently lower than those of SS, and peak grain filling also occurred later in IS than SS (**Figure 3**). Compared with treatment T0, treatment T2 significantly increased the initial and maximum grain filling rates of IS, and the peak value of the grain filling rate in W1844 was higher than that of SS in the T0 group. The changes in grain weight, grain filling rate, and sucrose content indicate that the removal of SS to force photosynthate transport to IS a feasible method for improving IS grain filling.

### Hormone Contents in Developing Grains

**FIGURE 4:**
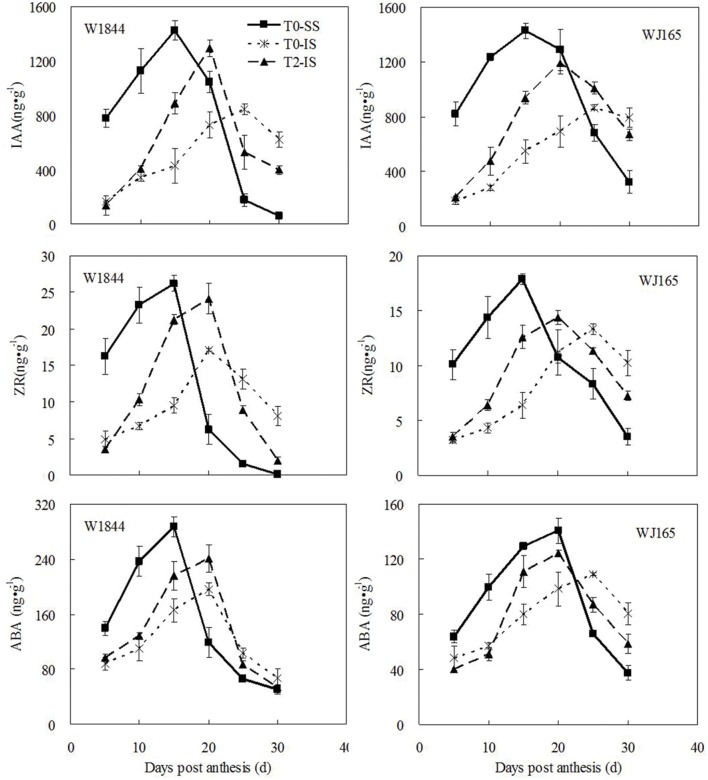
**Hormones content (ZR, IAA, and ABA) of SS and IS during the grain filling period of rice.** T0 and T2 represent control group without any treatment and top 2/3 of the spikelets were removed. Vertical bars represent mean values ± SE (*n* = 3).

As shown in **Figure 4**, there were large differences in the levels of the three hormones measured—ZR, IAA, and ABA—between SS and IS for W1844 during early grain filling (5–15 DPA), with higher levels in SS than in IS. For strain W1844, after 20 DPA, IS hormone levels were higher than those in SS. The highest hormone levels in SS and IS occurred at 15 and 20–25 DPA, respectively. T2 treatment significantly increased IS hormone levels in W1844. Compared with W1844, the highest hormone levels of SS and IS in WJ165 occurred a little later, and at 15–20 and 25 DPA, respectively. SS removal treatment significantly increased IS hormone levels in WJ165, and make them reaching the peak value 5 days in advance. In summary, after SS were removed, levels in IS of all three hormones responded by increasing. This indicates that improvements in IS grain filling may be achieved by elevating hormone levels.

### Activities of Key Enzymes Involved in Metabolism of Sucrose to Starch

**FIGURE 5:**
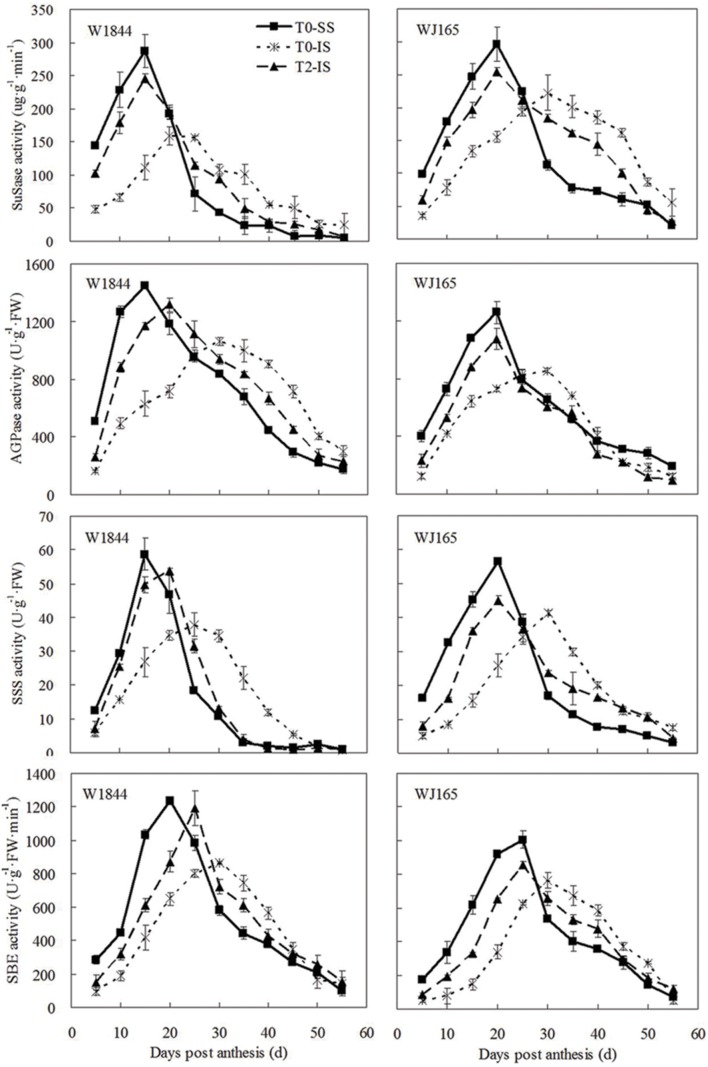
**Activities of key enzymes involved in sucrose to starch conversion in SS and IS during the grain filling period of rice.** T0 and T2 represent control group without any treatment and top 2/3 of the spikelets were removed. Vertical bars represent mean values ± SE (*n* = 3).

Similar to the changes we found in the grain filling rate, the SuSase activity change curve contained a single peak: enzyme activity at the beginning of the grain filling period was relatively low, followed by increased activity as grain filling progressed and then a rapid decrease (**Figure 5**). It took longer for enzyme activity to reach its peak in IS than in SS, and the peak value was also lower in IS. T2 treatment improved IS SuSase activity, and peak enzyme activity occurred earlier. Peak values of SuSase activity in IS under T2 treatment were 54.91% (W1844) and 14.28% (WJ165) higher than those in the T0 group. Although the activities of AGPase, SSS, and SBE reached their peak values at different times, the overall trends were similar to those of SuSase.

### Expression Levels of Genes Involved in Metabolism of Sucrose to Starch

**FIGURE 6:**
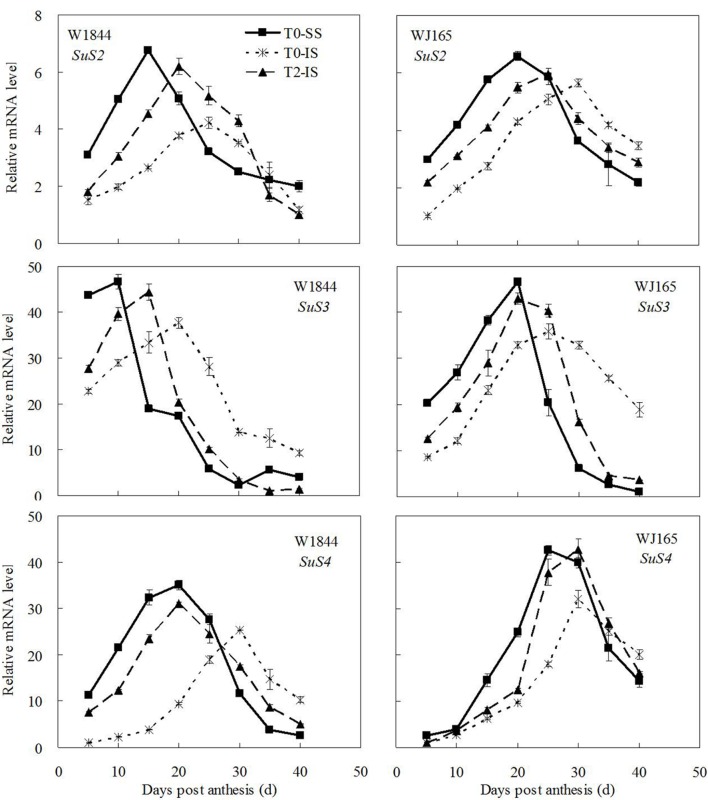
**Relative mRNA level of *SuS2*, *SuS3*, and *SuS4* in SS and IS during the grain filling period of rice.** T0 and T2 represent control group without any treatment and top 2/3 of the spikelets were removed. Vertical bars represent mean values ± SE (*n* = 3).

**Figure 6** shows that among the three isoenzymes of SuSase (*SuS2*, *SuS3*, and *SuS4*), only changes in the expression of *SuS2* were consistent with the changes in SuSase enzyme activity: relatively low in the early stage of the grain filling period, reaching a maximum during the middle of the filling stage, and then gradually declining thereafter (**Figure 5**). The peak of *SuS3* expression in W1844 appeared relatively early during the filling period, while the peak of *SuS4* expression in WJ165 appeared relatively late; both reflected slightly different expression dynamics than expected based on SuSase enzyme activity. Despite this, the relative expression levels of *SuS2*, *SuS3*, and *SuS4* in IS were all lower than those of SS, and spikelet thinning increased the expression levels of all three genes in IS. This indicates that these three genes respond to the spikelet-thinning treatment at the transcriptional level, thus regulating SuSase enzyme activity.

**FIGURE 7:**
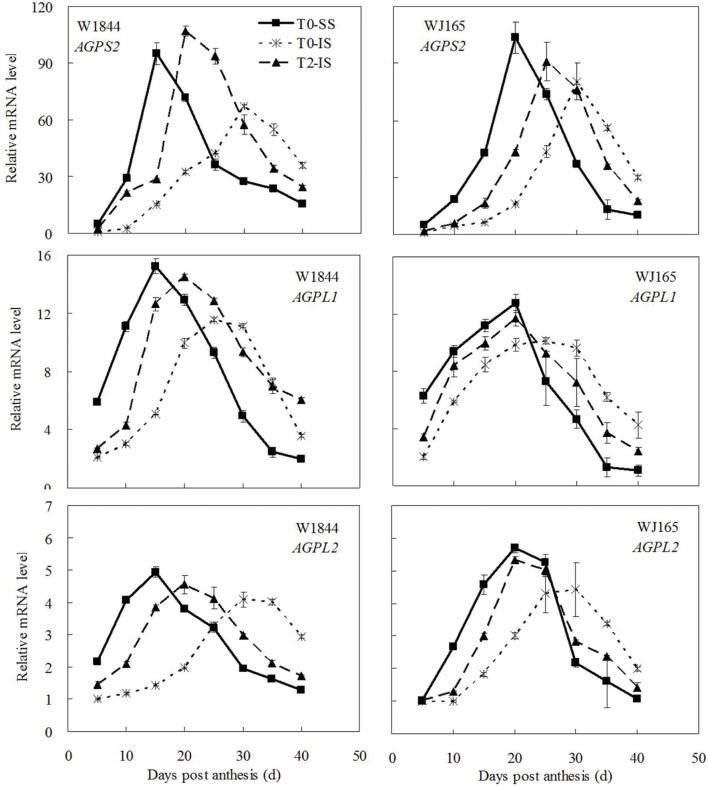
**Relative mRNA level of *AGPS2*, *AGPL1*, and *AGPL2* in SS and IS during the grain filling period of rice.** T0 and T2 represent control group without any treatment and top 2/3 of the spikelets were removed. Vertical bars represent mean values ± SE (*n* = 3).

**FIGURE 8:**
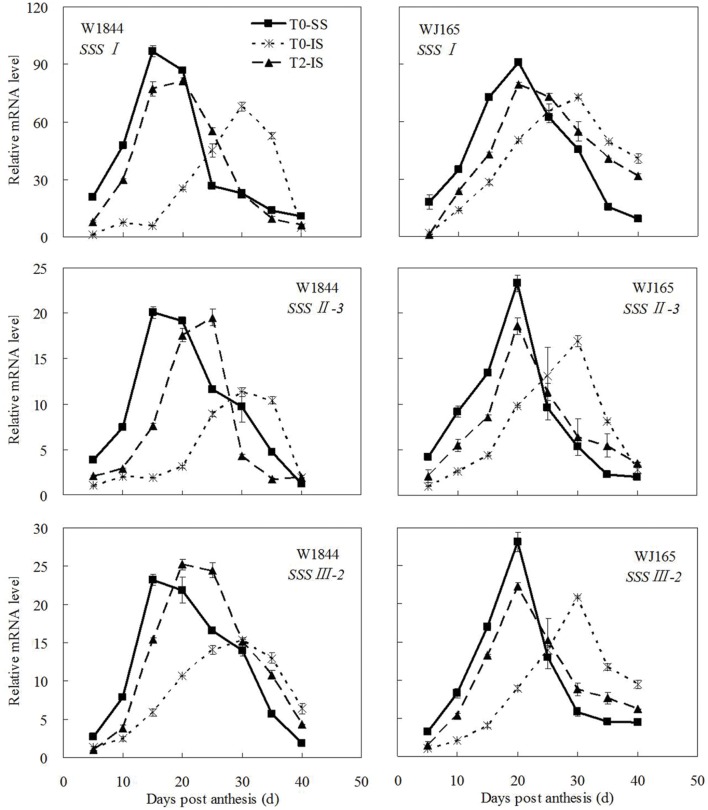
**Relative mRNA level of *SSSI*, *SSSII-3*, and *SSSIII-2* in SS and IS during the grain filling period of rice.** T0 and T2 represent control group without any treatment and top 2/3 of the spikelets were removed. Vertical bars represent mean values ± SE (*n* = 3).

**FIGURE 9:**
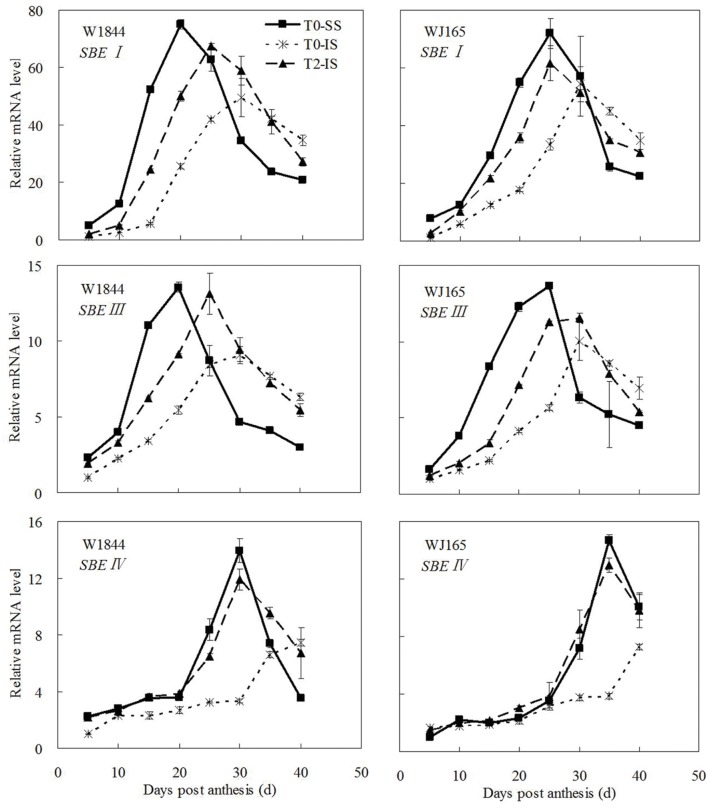
**Relative mRNA level of *SBEI*, *SBEIII*, and *SBEIV* in SS and IS during the grain filling period of rice.** T0 and T2 represent control group without any treatment and top 2/3 of the spikelets were removed. Vertical bars represent mean values ± SE (*n* = 3).

The genes *AGPS1*, *AGPL1*, *AGPL2*, *SSSI*, *SSSII-3*, *SSSIII-2*, *SBE I*, and *SBEIII* are all related to key starch synthesis enzymes. The changes in their expression levels were similar to those expected based on the changes in AGPase, SSS, and SBE enzyme activity and the trends in grain filling rates (**Figures 3**, **5**, and **7**–**9**). T2 treatment increased the expression levels of these genes in IS (**Figures 7–9**). In contrast, changes in the expression of *SBEIV* were not consistent with SBE enzyme activity and grain filling rates. At the early filling stage, *SBEIV* expression was relatively low, and its peak appeared late. However, its expression in IS was lower than that in SS. After spikelet thinning, *SBEIV* expression was up-regulated. In summary, after increasing the supply of assimilates to the IS, all genes encoding key starch synthesis enzymes responded with elevated expression levels.

Correlation analysis (**Figures 3** and **5**–**9**; **Supplementary Table S4**) showed that the enzyme activities of SuSase, AGPase, SSS, and SBE and the gene expression levels of *SuS2*, *SuS3*, *SuS4*, *AGPS1*, *AGPL1*, *AGPL2*, *SSSI*, *SSSII-3*, *SSSIII-2*, and *SBEIII* in W1844 (40 DPA) showed significantly positive correlations *R*^2^ = 0.405^∗^ (*P* < 0.05) to 0.920^∗∗^ (*P* < 0.01), while *SBEI* and *SBEIV* showed no significant correlation with the grain filling rate (*R*^2^ = 0.046 ∼ 0.153, *P* > 0.05). Similar results were also found in WJ165, indicating that following spikelet thinning, improvements in IS grain filling may be achieved via the regulation of key enzymatic activities and gene expression levels.

## Discussion

The phenomena of low seed setting rate and grain plumpness are common in large-panicle rice strains, and this is mainly due to poor IS grain filling and the formation of empty and blighted grains of rice (Yang and Zhang, 2006). This study also revealed a similar phenomenon: SS and IS of two large-panicle rice strains exhibited distinct differences in grain weight and grain filling rate. Many studies Ding et al. (2004), Yuan et al. (2004), and Fu et al. (2011) have shown that temperature could affect the grain filling of rice. Due to the different flowering dates between SS and IS, the grain filling of IS may be affected by the temperature. Analysis of meteorological data in 2014 (**Supplementary Table S1** and **Supplementary Figure S1**), we found that the daily mean temperature during flowering period of SS and IS were 25.4°C and 26.7°C respectively. Since daily mean temperature 25∼30°C is considered the optimum temperature for the flowering of rice, so temperature did not affect the flowering and pollination of inferior spikelets. Compared the daily mean temperature during grain filling period of SS and IS, we found that the daily mean temperature of IS was lower than that in SS, but due to its longer grain filling duration, the effective accumulated temperature of IS was higher than that in SS, therefore, in this study, temperature is not the main reason for the poor grain filling of IS.

Kobata et al. (2013) and Yoshinaga et al. (2013) suggested that in these high-yield cultivars, spikelet fertility contributes to poor grain filling. In this study, we found that SS removal significantly improved the IS seed setting rate in strains W1844 and WJ165. There are several possible reasons for this occurrence. First, inadequate assimilate supply around flowering results in spikelet sterility through a fault in the pollination process (Kobata et al., 2013); therefore, after SS removal, the number of fertilized grains increases with increases in the IS assimilate supply. Secondly, blighted IS grains transformed into plump grains because they obtained an adequate supply of assimilates. Comparing the pollen activities of SS, MS, and IS in strains W1844 and WJ165, we found that IS pollen activity was slightly lower than those of SS and MS, though all were higher than 90% (**Supplementary Table S2**). Therefore, spikelet sterility appears to have some impact on the IS seed setting rate, but this influence is relatively minimal and is not the primary cause of poor IS grain filling. In other words, under T2 treatment, the IS seed setting rate was improved, which can be largely attributed to increasing grain plumpness as the IS obtained a sufficient supply of carbohydrates.

Murty and Murty (1982), Venkateswarlu and Visperas (1987), Yuan (1997), and Cheng et al. (2003) proposed that the slow grain filling and low grain weight of IS were mainly due to limitations in the carbohydrate supply. Our current results support this theory, with the sucrose content, grain filling rate, and grain weight of IS increasing considerably upon SS removal (**Table 2**; **Figures 1–3**). Correlation analysis (**Supplementary Figure S2** and **Supplementary Table S4**) showed that the sucrose content and grain filling rate of W1844 and WJ165 (40 DPA) have a significantly positive correlations *R*^2^ = 0.688^∗∗^ and 0.526^∗∗^, respectively, which indicating that the improvement of grain filling in IS, when SS was thinning, might be achieved though the increasing of sucrose content in IS. Our findings regarding the sucrose content of grains differs from the results of Yang and Zhang (2010); this may be due to differences in the units chosen to represent sucrose content, as Yang and Zhang (2010) expressed sucrose content as mg g^-1^ DW, while we used μg grain^-1^. However, genotypic differences in the effect of SS removal on the development of IS in the rice panicle may be attributable to differences in panicle type or structure. Our results showed that T2 treatment increased the grain weight and grain filling rate of IS in both W1844 and WJ165, though the increase was higher for W1844, allowing IS to achieve similar values as those of SS in the T0 group (**Figures 1** and **2**). This may be due to grain growth density, as W1844 has a higher grain growth density than WJ165 (**Table 1**) and is therefore more sensitive to the effects of changes in the source/sink ratio. This corroborates the findings of Wang et al. (2006).

Plant hormone levels are closely associated with grain development, particularly during early grain filling. However, the effect of SS removal on hormone levels over the course of IS grain filling remains to be fully understood. Cytokinin (CTK) is involved in the regulation of cell division and cell elongation during early grain filling (Ozga and Reinecke, 2003). Yang et al. (2002, 2003) reported that cell numbers in the rice endosperm play a dominant role in determining grain weight and that poor IS grain filling can be mainly ascribed to their slow rates of cell division and elongation. In the current study, after SS removal, the IS ZR contents in both rice strains were higher than those of IS in the T0 treatment (**Figure 4**). ZR enhances IS endosperm cell division and elongation, thereby increasing the sink capacity of the IS.

IAA may represent the molecular signal by which IS development is inhibited by SS, and this SS inhibition may be reduced by exogenous IAA (Tian and Wang, 1998; Wang et al., 2001). Seth and Waering (1967) argued that IAA could control grain growth by regulating the distribution of assimilation products. The main role of IAA in grain filling is to increase the “pull” of their position to assimilates, so that assimilates are supplied primarily to locations with high IAA levels (Tian and Wang, 1998). This study, as well as several previous studies, found that the SS IAA content was much higher than that of the IS during early grain filling (**Figure 4**; Duan et al., 1999; Yang et al., 2003; Zhang et al., 2009). Consequently, assimilates are supplied primarily to the SS, and IS are unable to obtain a timely supply of nutrients after fertilization, resulting in a relative lag in IS grain filling. SS removal treatment, which severs the apical dominance of SS, results in an increased supply of assimilates to IS, thereby increasing IS grain filling.

ABA is another key determinant of grain filling (Kato and Takeda, 1993; Akihiro et al., 2006; Yang et al., 2006). Kato et al. (1993) and Brenner and Cheikh (1995) suggested that ABA may also be involved in the regulation of dry matter transfer and accumulation. Moreover, in the early grain filling stage, application of exogenous ABA to plants could significantly increase the remobilization of assimilates from the stem to the grains (Yang et al., 2004). The change of ABA concentration in the grains was consistent with the change of the grain filling rate (**Figures 3** and **4**). SS removing treatment significantly increased grain filling rate, grain weight and ABA content of IS. Correlation analysis showed that ABA content is positively associated with grain filling rate (Kato et al., 1993; Yang et al., 2006; Zhang et al., 2009), and such observations are consistent with those of previous reports involving wheat (Xie et al., 2003) and maize (Travaglia et al., 2012; Xu Y. et al., 2013). It is possible that insufficient ABA may be lead to the poor grain filling of IS. In short, after SS was removed, IS became a new growth center, and its content of ZR, IAA, and ABA was increased. Therefore, these hormones, in addition to its own physiological roles such as expanding sink capacity and promoting the growth of grains, its more important role is as a signals. IAA and ABA, for instance, which play a vital role in grain filling through prompting assimilates transfer to IS.

A large number of studies have indicated that the expression of genes encoding key starch synthesis enzymes is regulated by plant hormones. Exogenous application of ABA increases the gene expression of SuSase and its corresponding activity significantly (Tang et al., 2009; Zhu et al., 2011). Plant AGPase is a heterologous enzyme formed by two small subunits and four large subunits. The expression of AGPase is up-regulated by IAA and CTK (Miyazawa et al., 1999). In addition, Rook et al. (2001) and Akihiro et al. (2005) suggested that the expression of *OsAPL3* was induced and up-regulated by both exogenous ABA and sucrose. Many studies have shown that sucrose can induce the expression of the starch synthesis genes (Müller-Röber et al., 1990; Akihiro et al., 2005; Ahn et al., 2010). The expressions of the genes encoding SuSase, AGPase, SSS, and SBE are all up-regulated by sucrose (Salanoubat and Belliard, 1989; Müller-Röber et al., 1990; Hess and Willmitzer, 1996; Nielsen et al., 1998; Zeng et al., 1998; Ahn et al., 2010). In this study, we found that after spikelet thinning, as a result of increases in ZR, IAA, and ABA in IS, as well as an increase in the sucrose supply to the IS, the expression levels of genes encoding starch synthesis enzymes (i.e., *SuS2*, *SuS3*, *SuS4*, *AGPS1*, *AGPL1*, *AGPL2*, *SSSI*, *SSSII-3*, *SSSIII-2*, *SBEI*, *SBEIII*, and *SBEIV*) increased and IS grain filling subsequently improved.

In addition to enzyme regulation at the transcriptional level, the activities of key starch synthesis enzymes are also regulated by plant hormones. Yang et al. (2004), Zhu et al. (2011), and Wang et al. (2015) demonstrated that exogenous application of ABA during rice grain filling improved grain filling by regulating the activities of key enzymes involved in conversion of sucrose to starch. Studies have also shown that sucrose plays an important role in the regulation of starch synthesis enzyme activities. Guo and Xie (2013) found that exogenous sucrose treatment improved the activities of SuSase and starch phosphorylase (SPS), thus promoting the synthesis of chestnut starch. Ross and Davies (1992) placed potato tubers in solutions with different sucrose concentrations in an *in vitro* culture and found that SuSase activity in the tubers increased with increases in sucrose concentration of the culture medium. Tang et al. (2009) also observed that *in vitro* sugar treatment of the rice panicle could significantly increase SuSase activity, indicating that sucrose exerts a regulatory effect on SuSase activity. Our study, similar to the findings of some previous studies, found that the delayed initiation of grain filling and the low maximum filling rate of IS were closely correlated with low SuSase, AGPase, SSS, and SBE activities in the IS as compared with those of the SS (**Figure 5**; Takashi et al., 2005; Tatsuro et al., 2008; Wang et al., 2015). After spikelet thinning, the levels of ZR, IAA, and ABA in IS increased, as well as the sucrose supply to the IS increased, thereby enhancing the activities of SuSase, AGPase, SSS, and SBE (**Figures 3–5**). This led to increases in the IS grain weight and seed setting rate (**Table 2**). Therefore, poor IS grain filling in large-panicle rice may be closely related to insufficient assimilate supply and hormone imbalances.

After SS removal, the apical dominance of SS is released, leading to increased hormone levels (ZR, IAA, and ABA) in IS; this serves to expand the IS sink capacity and sucrose supply. Meanwhile, under this treatment, the expression and activities of key enzymes involved in starch synthesis are also elevated, thus further improving IS grain filling. Current research has only demonstrated that sucrose induces an elevation in SuSase activity, and there is little prior evidence of the effect of sucrose on the activities of AGP, SSS, and SBE. Therefore, more experiments are necessary to confirm the effect of sucrose on these enzymes. Further research is also needed to determine the mechanism of sucrose’s effect on enzyme activity in the starch synthesis pathway and the associated signaling/regulatory network.

## Conclusion

Rice grain filling is a complex and highly organized physiological and biochemical process that is highly regulated by environmental factors. There are substantial differences in the grain weight and grain filling rate of SS and IS in homozygous, large-panicle rice. SS removal was used to force photosynthate transport to the IS. Under this treatment, initiation of IS grain filling was promoted, the maximum grain filling rate was increased, and the IS grain weight was improved. SS removal treatment increased sucrose content and hormones levels of IS. This demonstrates that IS grain filling is restricted by SS through limitation of the assimilate supply and hormone imbalances. SS removal treatment improves IS grain filling primarily by increasing the IS sucrose supply and hormone levels, thus increasing the expression levels and activities of key enzymes involved in sucrose to starch metabolism.

## Author Contributions

CY, SW, and YD designed experiments; CY, BX, and WH collected samples; CY and HZ carried out experiments; CY analyzed experimental results. CD and ST assisted with semi-quantification analysis; CY and LC wrote the manuscript; ZL and GL modified the manuscript.

## Conflict of Interest Statement

The authors declare that the research was conducted in the absence of any commercial or financial relationships that could be construed as a potential conflict of interest.

## Abbreviations

ABA: abscisic acid
AGPase: ADP-glucose pyrophosphorylase
CTK: cytokinin
DPA: days post-anthesis
DW: dry weight
IAA: indole-3-acetic acid
IS: inferior spikelets
MS: medium spikelets
SBE: starch branching enzyme
SS: superior spikelets
SSS: soluble starch synthase
SuSase: sucrose synthase
ZR: zeatin riboside
