# Supplementary material for: Effect of Removing Superior Spikelets on Grain Filling of Inferior Spikelets in Rice
Source: Front Plant Sci. 2016 Aug 5;7:1161. doi: 10.3389/fpls.2016.01161 (PMC4974274; doi:10.3389/fpls.2016.01161)
Supplement: Supplementary file 1 [file Data_Sheet_1.DOCX]

**Supplementary Data**

Table 1. Daily mean temperature during flowing time and effective accumulated temperature during grain filling periods of Superior spikelets and Inferior spikelets in W1844 and WJ165.

| Materials | Spikelets  type | DMT during  flowing time (℃) | DMT during grain filling（℃） | Days of  Grain filling（d） | EAT  （℃） |
| --- | --- | --- | --- | --- | --- |
| W1844 | SS | 25.4 | 23.3 | 34 | 451.2 |
|  | IS | 26.7 | 21.3 | 51 | 573.9 |
| WJ165 | SS | 25.4 | 22.7 | 41 | 520.0 |
|  | IS | 26.7 | 21.3 | 49 | 553.8 |

SS-Superior spikelets; IS-Inferior spikelets; DMT - Daily mean temperature; EAT- Effective accumulated temperature.

Note: (1) Daily mean temperature (DMT) = (T_max_ + T_min_) / 2 (Andalesa et al., 2006; Confalonieri et al., 2004; Lu et al., 2001)

(2) Effective temperature = Daily mean temperature - Bottom growth temperature of rice (about 10℃ for japonica rice).

(3) Effective accumulated temperature (ETA) refers to the integral of effective temperature to time at a certain period, and the formula is as follows:

Table 2. Pollen activity of spikelets in different parts of the panicle.

| Materials | Superior spikelets | Medium spikelets | Inferior spikelets |
| --- | --- | --- | --- |
| W1844 | 95.90% a | 95.17% a | 91.76% b |
| WJ165 | 94.80% a | 93.48% a | 91.61% a |

The different lowercase letters labeled after the data from the same character under the same variety indicate significant differences at the 0.05 level.

Table 3. Sequence of primers for *Actin* and Genes of key enzymes involved in sucrose-to-starch conversion used for qRT-PCR.

| Gene  name | Accession No. | Up-primer(5’-3’) | Down-primer(5’-3’) |
| --- | --- | --- | --- |
| *Actin* | AK100267 | CAGCACATTCCAGCAGATGT | TAGGCCGGTTGAAAACTTTG |
| *SuS2* | AK072074 | GAGGCTGATGACCTTGACTGG | CCCTCCATTACTTGGATGTGCT |
| *SuS3* | AK100306 | TCAGCGCATCGAGGAGAAGT | GGCCAATGGAACGGTGGTAG |
| *SuS4* | AK102158 | TCCGTGAACTGGCGAAGACT | CCCAAGTTCGTCACTTGCTG |
| *OsAPS2* | AK103906 | TCTTTTGTTGCCCATTCATCTGG | TGATTCCAAGCACACTCTCATCGAC |
| *OsAPL1* | AK069296 | GGAAAGGTTCCTATTGGAATCG | GGAGGGCTTTATTCCACCTCAG |
| *OsAPL2* | AK071497 | TAGATAGGCCTTGGAATCGCACC | TAGAGTTCCCATTCCAAAACAAACC |
| *SSSⅠ* | D16202 | CTCTTCGTGGTCATCGTGT | GCAATCCCAGGTCAGGATA |
| *SSSⅡ-3* | AF419099 | AGGTGGGCTTGGAGATGTT | CAGATAAACAGGCAGGAGTG |
| *SSSⅢ-2* | AY100469 | CGCAGGACTTTACGACAC | CTCTTGCCACCGTTGGAT |
| *SBEⅠ* | AK065121 | CGAGGGAATGCCAGGAGT | GCGATAGTAAGCCACACAGGT |
| *SBEⅢ* | AB023498 | AGCCAGGGAAATATAAGGTGGT | GTCATGTGAACAATCGGCAG |
| *SBEⅣ* | D16201 | CTCACGTGTAAAGATTCGGATG | GTATTTCACCTGGAGCCTGC |

Table 4. Correlations of grain filling rate with content of sucrose as well as the activities and gene expression levels of the enzymes involved in sucrose to starch conversion in rice spikelets during the first 40 days after anthesis.

| Correlations with | Grain filling rate | |
| --- | --- | --- |
|  | W1844 | WJ165 |
| Content of sucrose | 0.688** | 0.526** |
| Activity of enzymes |  |  |
| Sucrose synthase | 0.785** | 0.758** |
| ADP-glucose pyrophosphorylase | 0.625** | 0.763** |
| Soluble starch synthase | 0.768** | 0.792** |
| Starch branching enzyme | 0.346** | 0.672** |
| Expression levels of the genes |  |  |
| *Sucrose synthase 2 (SuS2)* | 0.847** | 0.892** |
| *Sucrose synthase 3 (SuS3)* | 0.186* | 0.535** |
| *Sucrose synthase 4 (SuS4)* | 0.739** | 0.382** |
| *ADP-glucose pyrophosphorylase small subunit 2 (AGPS2)* | 0.446** | 0.707** |
| *ADP-glucose pyrophosphorylase large subunit 1 (AGPL1)* | 0.706** | 0.577** |
| *ADP-glucose pyrophosphorylase large subunit 2 (AGPL2)* | 0.677** | 0.860** |
| *Soluble starch synthase Ⅰ (SSSⅠ)* | 0.732** | 0.849** |
| *Soluble starch synthase II-3 (SSSII-3)* | 0.588** | 0.676** |
| *Soluble starch synthase Ⅲ-2 (SSSⅢ-2)* | 0.530** | 0.681** |
| *Starch branching enzyme I (SBEI)* | 0.153 | 0.571** |
| *Starch branching enzyme Ⅲ (SBEⅢ)* | 0.308** | 0.644** |
| *Starch branching enzyme Ⅳ (SBEⅣ)* | 0.046 | 0.033 |

R^2^_0.05_=0.164; R^2^_0.01_=0.265; *, ** Correlation significance at P = 0.05 and P = 0.01 levels, respectively.

Fig. 1. Temperature during the grain filling period of rice in 2014 at the experiment site of DanYang, Southeast China. Data are means of per 24 hours from the heading of rice.

Fig. 2. Correlations between grain filling rate and sucrose content of W1844 and WJ165 during the first 40 days after anthesis.
